# Study on Basic Pavement Performance of High-Elasticity Asphalt Concrete

**DOI:** 10.3390/polym16152156

**Published:** 2024-07-29

**Authors:** Juan Wang, Taixu Huo, Dahui Wang, Peng Zhang

**Affiliations:** 1 School of Water Conservancy and Transportation, Zhengzhou University, Zhengzhou 450001, China; wangjuan@zzu.edu.cn (J.W.);; 2 Henan Key Laboratory of Safety Technology for Water Conservancy Project, Henan Provincial Water Conservancy Technology Application Center, Zhengzhou 450003, China

**Keywords:** elastic asphalt concrete, rubber particles, polyester fibers, pavement performance, deformation recovery performance

## Abstract

In order to improve the basic pavement performance of high-elastic asphalt concrete filled in the expansion longitudinal joints of seamless bridges, rubber particles and polyester fibers were added to optimize the mix proportion of elastic asphalt concrete, and the optimal asphalt–aggregate ratio was determined. The influence of rubber particles and polyester fibers on the basic pavement performance of high-elastic asphalt concrete was studied. The results show that when the dosage of polyester fiber is not more than 0.6%, the optimal asphalt–aggregate ratio is 1:5, and when it exceeds 0.6%, the optimal asphalt–aggregate ratio is 1:4. The incorporation of rubber particles reduces the compressive strength of high-elastic asphalt concrete but enhances its high-temperature stability, fracture performance, and deformation recovery ability. The incorporation of polyester fibers improves its compressive strength, high-temperature stability, fracture performance, and deformation recovery ability. In addition, the incorporation of rubber granules and polyester fibers promotes the use of green building materials and provides strong support for sustainable building practices.

## 1. Introduction

The bridge widening longitudinal joint is an important structure during bridge widening, which mainly aims to satisfy the deformation of the bridge deck and ensure smoothness of the bridge deck and comfortable driving [1]. The main working mechanism of seamless bridge expansion longitudinal joint relies on the elastic–plasticity of high elastic asphalt mixture to adapt to the deformation of the bridge deck caused by the uneven displacement of new and old bridges, the repeated effect of vehicle and temperature load.

Due to its special working characteristics, seamless bridge expansion longitudinal joints are prone to rutting and cracking. To ensure the performance of expansion joints, modified asphalt concrete should have excellent pavement performance and deformation recovery performance to meet the requirements of bridge widening longitudinal joints. Scholars usually add polyphosphoric acid [2], biochar [3], nanomaterials (such as nano-silica powder [4], carbon nanotubes [5], nano-calcium carbonate [6], nano-iron oxide [7], etc.), rubber particles [8,9,10,11], and polymer fibers to improve the high-temperature performance [12,13,14,15], crack resistance, and fatigue performance of asphalt concrete. Xie added 20% rubber powder to SBS-modified asphalt to prepare a seamless expansion joint binder with improved high and low-temperature performance. SBS is a triblock copolymer with styrene and butadiene as monomers, and is a thermoplastic elastomer. By adding rubber particles to prepare asphalt concrete, it was found that asphalt concrete has better toughness at low temperatures [16]. Yu modified asphalt mixtures with single and composite rubber modifiers and found that the low-temperature performance of asphalt concrete was improved under both schemes [17]. Shu conducted a secondary modification of SBS-modified asphalt by adding rubber particles and modified asphalt concrete by adding rubber particles externally. He found that its low-temperature crack resistance was significantly increased, and its flexibility performance was excellent [18]. Xie prepared asphalt binder using AH70 asphalt with 7% SBS modifier, rubber powder, anti-rutting agent, and light oil, discovering that it exhibited excellent low-temperature flexibility, storage stability, and balanced high–low-temperature performance, ensuring that the single-sized asphalt concrete met project standards [19]. Zhan successively added rubber oil, rubber powder, SBS, and toughening agent to 70 base asphalt to prepare high-elastic modified asphalt. When no filler was added, its performance was basically equivalent to commonly used seamless expansion joint binders abroad. After being prepared into asphalt concrete, its high-temperature stability and low-temperature crack resistance were comparable to foreign products, showing excellent toughness [20]. This paper enhances the high-temperature stability and durability of asphalt concrete materials by mixing rubber particles and polyester fibers to improve the service life of roads, bridges, and other infrastructures, promote the application of green building materials, and provide strong support for sustainable building practices.

This article uses single-particle graded aggregates mixed with rubber particles and polyester fibers to prepare high-elastic asphalt concrete. Through experimental testing and analysis of its compressive strength, high-temperature stability, low-temperature crack resistance, deformation recovery performance, etc., it provides certain engineering technical guidance for the use of modified asphalt-filled bridge expansion joints.

## 2. Experimental Preparation

### 2.1. Materials

Bridge splicing wide longitudinal joints filled with asphalt concrete must have high elasticity and high crack resistance; BJ200 asphalt has an excellent softening point and elastic recovery rate, and it belongs to the high elasticity asphalt, so for this article, BJ200 asphalt was selected as the binder for bridge expansion joint mixture, and its basic properties are shown in Table 1.

Basalt with a single particle size distribution, which has a particle size of 5–10 mm, was chosen. Single-sized aggregates are embedded within each other to form a skeleton. Due to the absence of fine aggregates and mineral powder, the voids between these aggregates can accommodate a high content of asphalt binder, thereby taking into account both the strength and deformation adaptability of the asphalt concrete.

In addition, this paper utilized 1–3 mm rubber particles derived from crushed waste tires at dosages of 2%, 3%, and 4% to address the issue of excessive waste tires to a certain extent, and its basic properties are shown in Table 2. The rationale behind selecting these rubber particles is twofold: firstly, excessively large rubber particles are not suitable for the compaction of asphalt concrete, as they reduce adhesion with the asphalt and increase the rebound effect, leading to more serious initial defects within the asphalt concrete’s internal structure, which is detrimental to structural safety. Secondly, an excessive amount of rubber particles mixed into the asphalt concrete can destabilize its internal spatial skeleton, adversely affecting its overall performance.

Lastly, polyester fibers of 6 mm length at dosages of 0.6%, 1.2%, and 1.8% were added, and their basic performance parameters are shown in Table 3. The bridge longitudinal joints filled with asphalt concrete require sufficient deformation adaptability; therefore, rigid fibers cannot be selected. Compared to other fibers, polyester fibers exhibit superior performance in terms of non-agglomeration and crack resistance, and their low cost makes them cost-effective. Furthermore, when dispersed in three dimensions within the asphalt concrete, the fibers form a network of entangled constraints that strengthen the mixture. The large specific surface area of polyester fiber, coupled with its strong adsorption capacity, helps maintain the overall structure of the asphalt concrete, effectively enhancing its various properties and making it less prone to cracking. This paper aims to determine the optimal fiber content for the splicing of bridge expansion projects. When the length of the polyester fiber is 6 mm, it exhibits good adhesion with asphalt and can effectively enhance the mechanical properties of asphalt concrete. Furthermore, research indicates that the fiber content in fiber-reinforced asphalt should not exceed 8.3% of the asphalt mass. Based on this, and considering the oil-to-stone ratio selected in this paper, calculations were performed to determine the mixing amounts of the polyester fiber at four levels: 0%, 0.6%, 1.2%, and 1.8%, respectively.

### 2.2. Test Method

According to the specifications [21,23], the compressive strength, low-temperature crack resistance, high-temperature stability, and compression elastic recovery rate of high-elastic asphalt concrete were tested. The universal testing machine is used for compressive, fracture, and compressive elastic recovery tests, and the rutting test is selected for high-temperature stability.

The compression and rutting tests were conducted strictly in accordance with the specifications; the standard Marshall specimen was selected for the compression elastic recovery test, with the test temperature set at 20 °C. The standard Marshall specimen is a cylindrical specimen with a diameter of 101.6 mm and a height of 63.5 mm, which is formed by compacting with a compactor. During the test, the compression thickness of the specimen was controlled at approximately 5% of the original specimen thickness. After unloading, the specimen was placed in a 20 °C environment for 30 min to recover, and the thickness of the specimen before and after the test was measured. The compression elastic recovery rate of high-elastic asphalt concrete is calculated through Equation (1):

(1) $$D=\frac{\Delta h-\left(h_{0}-h_{i}\right)}{\Delta h}\times 100\%$$

where D is the compression elastic recovery rate; h_i_ is the thickness of the sample after compression of 5% and recovery; h_0_ is the original thickness of the sample; Δh is the compression amount of the sample, specifically 5% of the thickness of the sample.

The fracture test adopts the Semicircular Bend (SCB) fracture test, which is cut from a Marshall specimen with a thickness of 30 mm. The sample is cut along the symmetrical half-axis, with a length of 10 mm and a width not exceeding 1.5 mm as the initial crack. The experimental temperature was 0 °C, and the fracture toughness and fracture energy are calculated through Equations (2)–(6) [23]:

(2) $$K_{\mathrm{IC}}=\frac{P_{\max}}{2\mathrm{rt}}\sqrt{\pi a}Y$$

(3) $$Y=4.782+1.219\left(\frac{a}{r}\right)+0.063\exp\left(7.045\left(\frac{a}{r}\right)\right)$$

(4) $$G_{f}=\frac{W_{f}}{A_{\mathrm{lig}}}$$

(5) $$W_{f}=\int \mathrm{Pdu}$$

(6) $$A_{\mathrm{lig}}=\left(r-a\right)\times t$$

where K_IC_ is the fracture toughness; r is the radius of the specimen; a is the length of the specimen incision; t is the thickness of the specimen; P_max_ is the peak load; Y is the normalized stress intensity factor; G_f_ is the fracture energy; W_f_ is the fracture work; A_lig_ is the area of the toughness zone; P is the load; u is displacement.

### 2.3. Determine the Optimal Asphalt Content

The amount of asphalt binder is determined using the Marshall test method. Due to its significantly higher asphalt content compared to ordinary asphalt concrete, the high-temperature stability of this material is of utmost importance. Marshall stability can characterize its high-temperature performance. Therefore, Marshall stability is selected as the optimal control index for asphalt content. Based on existing engineering experience, the ratio of elastic asphalt concrete asphalt to aggregate used in seamless expansion joints is mostly around 1:5. This article conducted Marshall tests on rubber particles and polyester fiber asphalt concrete using oil aggregate ratios of 1:4, 1:5, 1:6, and 1:7.

**Figure 1:**
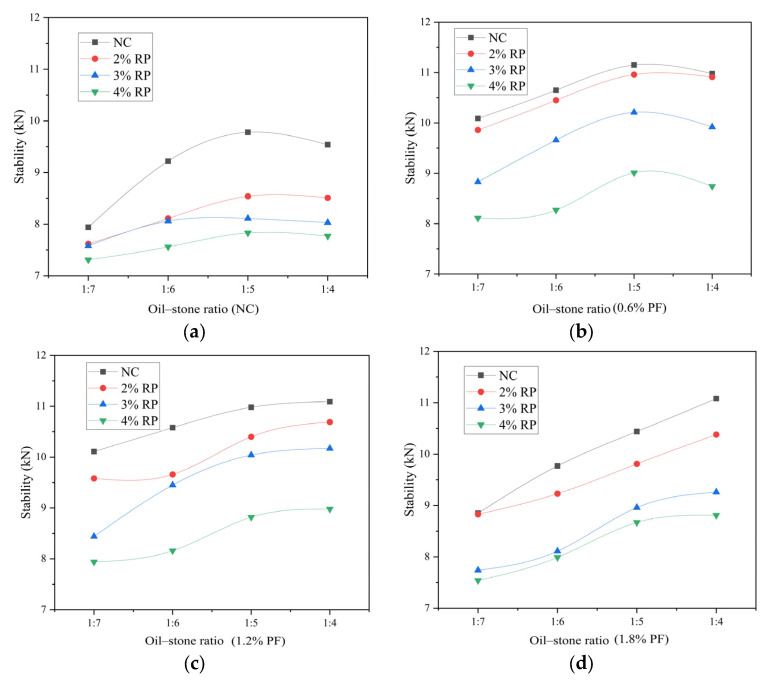
Result of Marshall stability: (**a**) This is a plot of the Marshall stability results for each proportion at each oil/stone ratio without polyester fiber mixing. (**b**) This is a plot of the Marshall stability results for each proportion at each oil/stone ratio with 0.6% polyester fibers mixing. (**c**) This is a plot of the Marshall stability results for each proportion at each oil/stone ratio with 1.2% polyester fibers mixing. (**d**) This is a plot of the Marshall stability results for each proportion at each oil/stone ratio with 1.8% polyester fibers mixing. NC stands for baseline bituminous concrete, RP stands for rubber particles and PF stands for polyester fiber.

The stability and flow values of high elastic asphalt concrete under different ratios are shown in Figure 1. The stability range of high elastic asphalt concrete is between 7.0 kN and 12 kN, which is lower than the Marshall stability of current road asphalt concrete, indicating that the prepared elastic asphalt concrete has significant flexibility. As the content of rubber particles increases, the Marshall stability of asphalt concrete gradually decreases. This is because rubber particles are added to asphalt concrete in the form of aggregates. Due to the high elasticity and low strength characteristics of rubber particles, the internal structure of asphalt concrete is affected by the rebound effect of rubber particles during the placement stage after compaction, leading to deterioration of the internal structure of asphalt concrete and decreased stability.

On the other hand, with the increase in polyester fiber content, the Marshall stability of asphalt concrete first increases and then decreases. Polyester fibers have a large specific surface area and good lipophilicity, providing a good wetting interface for asphalt and enhancing its adsorption capacity. An appropriate amount of polyester fiber is evenly distributed in the asphalt binder and is not prone to clumping. Polyester fibers are completely wrapped in asphalt, and they play a reinforcing role in asphalt concrete, significantly improving its strength.

In addition, when the polyester fiber content does not exceed 0.6%, the Marshall stability first increases and then decreases with the increase in asphalt content. When the oil–stone ratio is 1:5, the Marshall stability reaches the highest level. When the polyester fiber content exceeds 0.6%, the Marshall stability gradually increases with the increase in the oil–stone ratio. When the oil–stone ratio is 1:4, the Marshall stability reaches its highest. When the asphalt content is relatively low, the asphalt is not sufficient to fill the gaps between individual particle-size skeletons, resulting in an unstable skeleton structure and lower stability. As the oil-to-stone ratio increases, the voids are gradually filled, and the stability begins to improve. The filling of polyester fibers can adsorb more asphalt binder, so with the addition of polyester fibers, the corresponding oil–stone ratio gradually increases at the highest Marshall stability.

**Figure 2:**
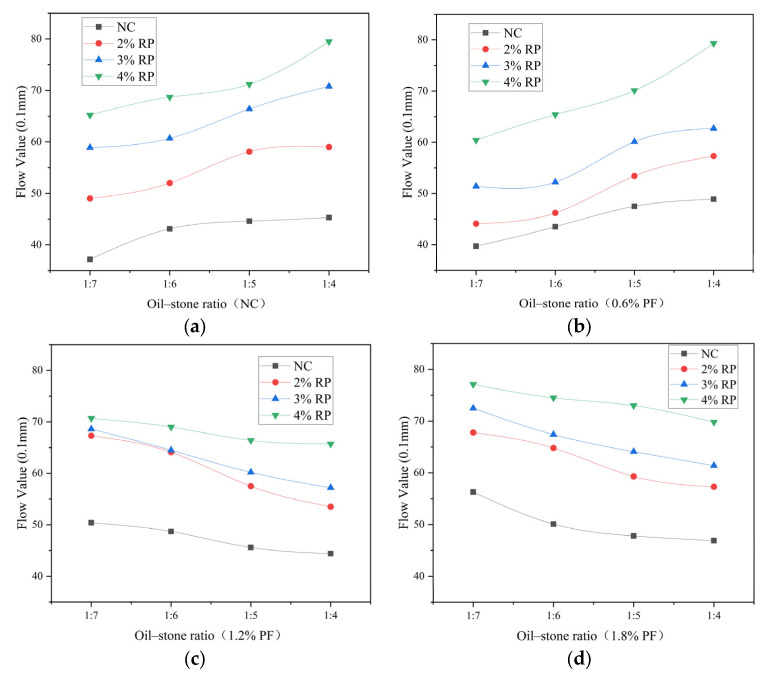
Result of flow value: (**a**) This is a plot of the flow value results for each proportion at each oil/stone ratio without polyester fiber mixing. (**b**) This is a plot of the flow value results for each proportion at each oil/stone ratio with 0.6% polyester fibers mixing. (**c**) This is a plot of the flow value results for each proportion at each oil/stone ratio with 1.2% polyester fibers mixing. (**d**) This is a plot of the flow value results for each proportion at each oil/stone ratio with 1.8% polyester fibers mixing.

The results of the flow values for each ratio are shown in Figure 2. As the rubber particle content increases, the flow value of asphalt concrete gradually increases. When the polyester fiber content does not exceed 0.6%, the flow value gradually increases with the increase in the oil–stone ratio, and the asphalt content has a major impact on the flow value results. When the polyester fiber content exceeds 0.6%, the flow value gradually decreases with the increase in the oil–stone ratio. At this fiber content, when the oil–stone ratio is 1:7, the fiber content has exceeded the optimal fiber content corresponding to the oil–stone ratio. Fiber clumping reduces the bonding between asphalt and aggregates, leading to internal deterioration of the concrete, resulting in the highest corresponding flow value. When the oil-to-stone ratio increases, the flow value decreases, but the decrease is not significant, and the effect of fiber reinforcement begins to show.

The high oil-to-stone ratio of elastic asphalt concrete used in bridge longitudinal joint splicing makes it significantly higher than ordinary asphalt concrete, thus resulting in poor high-temperature stability. In determining the optimal oil-to-stone ratio, the ratio that corresponds to the highest Marshall stability among each group of ratios is selected as the optimal one. According to the results of the Marshall test, when the mixing amount of polyester fiber is not more than 0.6%, the corresponding optimal oil-to-stone ratio is 1:5, whereas when the mixing amount of polyester fiber exceeds 0.6%, the corresponding optimal oil-to-stone ratio is 1:4. The mix compositions used in subsequent tests are presented in Table 4.

## 3. Results and Discussion

### 3.1. Compressive Performance

**Figure 3:**
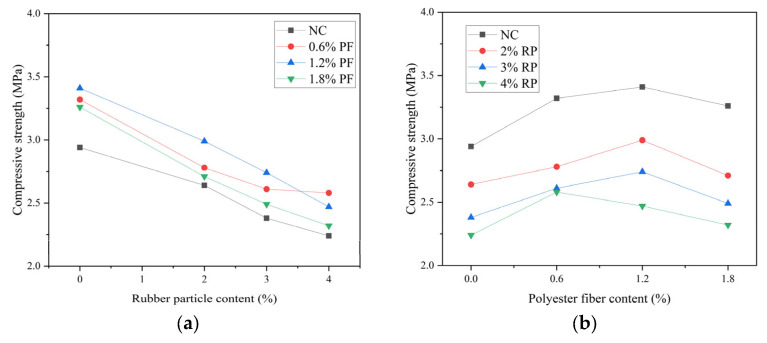
Result of compressive strength: (**a**) This is a figure of the compressive strength results of asphalt concrete when rubber granules are the variable. (**b**) This is a figure of the compressive strength results of asphalt concrete when polyester fibers are the variable.

The compressive strength of high-elastic asphalt concrete is shown in Figure 3. According to most researchers, the compressive strength of asphalt concrete at 20 °C typically ranges from 2.5 MPa to 5 MPa. However, due to the high asphalt content in this study, its compressive strength is relatively low. In actual projects, the compressive strength of asphalt concrete used to fill bridge spliced wide longitudinal joints is generally required to exceed 2 MPa. Nevertheless, the compressive strength of the high-elasticity asphalt concrete studied here meets these requirements. When rubber particles are added alone, the compressive strength gradually decreases with the increase in rubber particles. When the rubber particle content is 2%, 3%, and 4%, the compressive strength decreases by 10.2%, 19.0%, and 23.8%, respectively. When polyester fiber is added alone, with the increase in polyester fiber content, the compressive strength first increases and then decreases. When the polyester fiber content is 0.6%, 1.2%, and 1.8%, the compressive strength increases by 12.9%, 16.0%, and 10.9%, respectively. The maximum improvement in compressive strength is achieved when the fiber content is 0.6%. When polyester fibers and rubber granules were added in combination, the compressive strength of the asphalt concrete was lower than that of asphalt concrete without either polyester fibers or rubber granules, with the exception of the R2F1.2 combination group, which exhibited a 1.7% increase in compressive strength. As evident from the compounding results, the impact of rubber particles on compressive strength surpasses that of polyester fibers.

The addition of rubber particles changes the original dense structure of asphalt concrete. The strength of asphalt concrete mainly depends on the mutual embedding and internal friction between aggregates. Due to the low hardness and high elasticity of rubber particles themselves, they shrink and deform under load, which can easily cause changes inside the concrete. An appropriate amount of polyester fiber plays a reinforcing role in concrete. Polyester fibers are wrapped in asphalt binder, allowing asphalt concrete to disperse the load onto aggregates and asphalt binder in a timely manner after loading, making the stress on asphalt concrete more uniform and improving its strength.

### 3.2. High Temperature Stability

**Figure 4:**
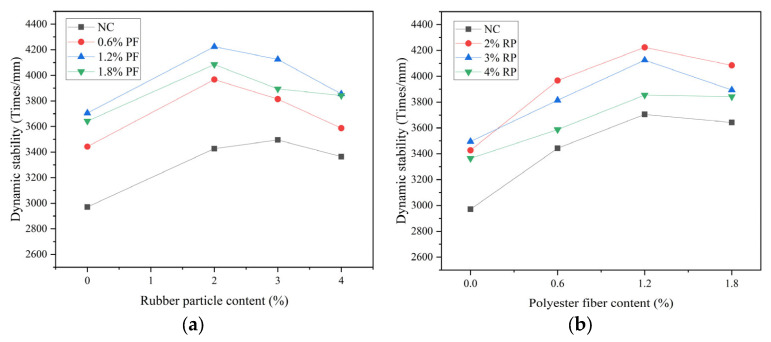
Result of dynamic stability: (**a**) This is a figure of the dynamic stability results of asphalt concrete when rubber granules are the variable. (**b**) This is a figure of the dynamic stability results of asphalt concrete when polyester fibers are the variable.

The dynamic stability of high-elastic asphalt concrete is shown in Figure 4. The dynamic stability of each ratio group is above 2900 cycles/mm, which meets the requirements of relevant Chinese specifications [21]. This paper is prepared with a focus on modified asphalt concrete in accordance with the requirements of the Chinese specifications, which stipulate that its dynamic stability must be higher than 2800 times/mm. When using unadulterated rubber particles and polyester fibers, its performance barely meets the requirements. After modification, its performance was greatly improved, meeting the requirements for the widened section of bridge splicing. When rubber particles are added alone, the dynamic stability first increases and then decreases with the increase in rubber particles. When the dosage is 2%, 3%, and 4%, the dynamic stability increases by 15.3%, 17.6%, and 13.2%, respectively. When polyester fibers are added alone, the dynamic stability first increases and then decreases with the increase in polyester fibers. When the content is 0.6%, 1.2%, and 1.8%, the dynamic stability increases by 15.9%, 24.7%, and 22.6%, respectively. The maximum improvement in dynamic stability of high elastic asphalt concrete is achieved when the fiber content is 1.2%. Compared with the dynamic stability results of benchmark asphalt concrete, the dynamic stability of each composite ratio group is higher than that of benchmark asphalt concrete when mixed with polyester fibers and rubber particles. The R4F0.6 group showed the lowest improvement in dynamic stability, at 20.7%. The R2F1.2 group has the best high-temperature stability, with a 42.2% increase in dynamic stability. Overall, the appropriate addition of rubber particles and polyester fibers can effectively improve the high-temperature stability of asphalt concrete.

The admixture of rubber particles created a spring-like structure within the asphalt concrete. As the test began, the asphalt concrete was compressed to produce rutting, and the rubber particles were also compressed and contracted accordingly. The elastic resilience of the rubber particles provided kinetic energy for the asphalt concrete to resist rutting. Polyester fibers existed in the mixture as a three-dimensional dispersed phase, forming a crisscross network structure that played a reinforcement role. Together with asphalt cement, the fibers strengthened the restraint on the framework and limited the lateral displacement of the aggregate, thus enhancing the high-temperature stability of the asphalt concrete [24].

### 3.3. Deformation Recovery Performance

**Figure 5:**
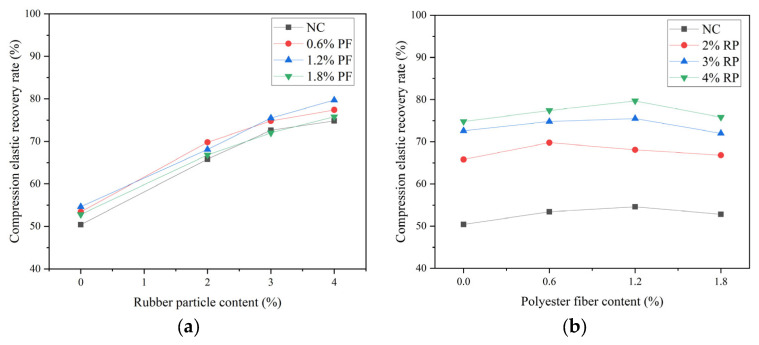
Result of compression elasticity recovery rate: (**a**) This is a figure of the compression elasticity recovery rate results of asphalt concrete when rubber granules are the variable. (**b**) This is a figure of the compression elasticity recovery rate results of asphalt concrete when polyester fibers are the variable.

The compression elastic recovery rate of high-elasticity asphalt concrete is shown in Figure 5. When rubber particles were added alone, the compression elastic recovery rate gradually increased with the increase in rubber particles. When the dosages were 2%, 3%, and 4%, the compression elastic recovery rate increased by 31.7%, 43.3%, and 49.4%, respectively. When polyester fibers were added alone, the compression elastic recovery rate first increased and then decreased with the increase in polyester fibers. When the dosages were 0.6%, 1.2%, and 1.8%, the compression elastic recovery rate increased by 6.0%, 7.3%, and 5.8%, respectively. The maximum increase in compression elastic recovery rate occurred when the fiber content was 1.2%. In the case of the mixed addition of polyester fibers and rubber particles, compared with the compression elastic recovery rate of the base asphalt concrete, the compression elastic recovery rate of each mixed addition group was higher than that of the base asphalt concrete. Among them, the R2F1.8 group had the lowest increase rate, which was 32.5%, while the R4F1.2 group had the highest increase rate, which was 58.1%. Overall, under the mixed addition conditions, the influence of rubber particles on the deformation recovery ability of high-elasticity asphalt concrete was far greater than that of polyester fibers.

The addition of rubber particles reduces the overall deformation of asphalt concrete, increases the resistance of asphalt mixture to deformation, and also enhances its recovery ability. Under the condition of controlling the compression deformation, its original skeleton structure is not severely damaged. When it is compressed, the rubber particles filled in the void of aggregate are also compressed and contracted. After the load is unloaded, the rubber particles extend in all directions, and the large elastic recovery ability of the rubber particles provides certain kinetic energy for the recovery of asphalt concrete. The admixture of polyester fibers plays a role in reinforcement in asphalt concrete. The fibers are wrapped in asphalt cement, and after the mixture is compressed and unloaded, they also have a certain impact on the deformation recovery of the asphalt mixture.

### 3.4. Low-Temperature Crack Resistance Performance

**Figure 6:**
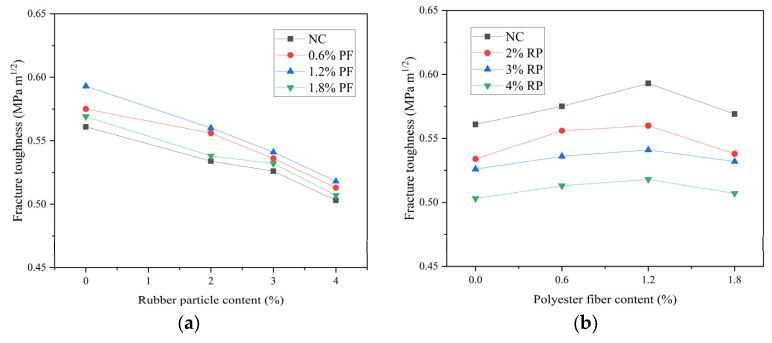
Result of fracture toughness: (**a**) This is a figure of the fracture toughness results of asphalt concrete when rubber granules are the variable. (**b**) This is a figure of the fracture toughness results of asphalt concrete when polyester fibers are the variable.

**Figure 7:**
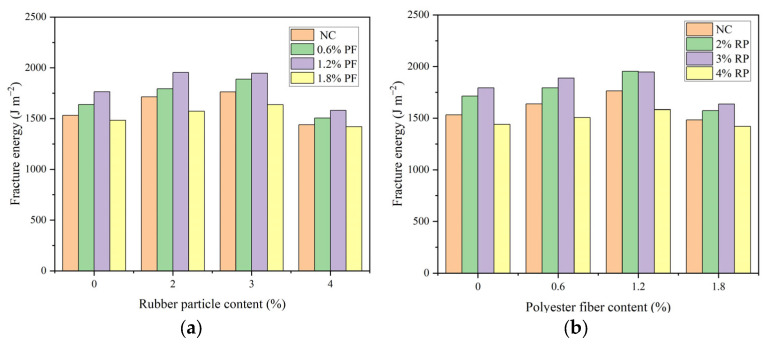
Result of fracture energy: (**a**) This is a figure of the fracture energy results of asphalt concrete when rubber granules are the variable. (**b**) This is a figure of the fracture energy results of asphalt concrete when polyester fibers are the variable.

The results of the fracture toughness and fracture energy of high-elasticity asphalt concrete are shown in Figure 6 and Figure 7. As can be seen from Figure 6 and Figure 7, when rubber particles are added alone, with the increase in rubber particles, the fracture toughness gradually decreases, and the fracture energy first increases and then decreases. When the dosage of rubber particles is 2%, 3%, and 4%, the fracture toughness decreases by 4.8%, 6.3%, and 10.3% compared with that without rubber particles, and the fracture energy increases by 11.8%, 15.0%, and −6.1%, respectively. When polyester fibers are added alone, with the increase in polyester fibers, the fracture toughness and fracture energy first increase and then decrease. When the dosage of polyester fibers is 0.6%, 1.2%, and 1.8%, the fracture toughness increases by 2.5%, 5.7%, and 1.3% compared with that without polyester fibers, and the fracture energy increases by 6.8%, 15.1%, and −3.2%, respectively. When rubber particles and polyester fibers are mixed, with the dosage of rubber particles at 2% and polyester fibers at 1.2%, the fracture toughness and fracture energy are the highest. Among them, the fracture toughness changes little compared with the base asphalt concrete, and the fracture energy increases by 27.5%.

As rubber particles were used to replace aggregates in high-elasticity asphalt concrete, the overall stiffness of the high-elasticity asphalt concrete decreased, resulting in a reduction in peak load. The calculation of fracture toughness was only related to the specimen size and peak load. When the specimen size was consistent, the fracture toughness value only depended on the peak load, and thus, the fracture toughness also decreased. During the fracture process, because the rubber particles bonded closely with the asphalt binder when the crack extended to the rubber particles, the rubber particles would be stretched, absorbing and dissipating energy until the rubber particles detached from the asphalt binder. Therefore, the incorporation of rubber particles increased the fracture energy. Polyester fibers were distributed in a three-dimensional network within the asphalt concrete, and their strength and stiffness were usually greater than those of the asphalt concrete. When cracks reached the fibers, the load was borne by the fibers. The anchoring effect of the fibers prevented the development of cracks. When additional load was applied, the stress concentration at the crack tip caused the fibers to detach from the asphalt binder or break. Since the fibers themselves had high tensile strength, this increased the material’s fracture strength. Although the incorporation of rubber particles reduced the strength of high-elasticity asphalt concrete, it enhanced its fracture performance. The reduced strength could be improved by adding polyester fibers. A suitable amount of polyester fibers enhanced both the strength and deformation performance of high-elasticity asphalt concrete. The combined use of both materials further improved the low-temperature cracking resistance of high-elasticity asphalt concrete.

## 4. Conclusions and Outlook

For bridge longitudinal joints filled with highly elastic asphalt concrete, its crack resistance and deformation performance are particularly crucial. Considering the compressive strength, high- and low-temperature performance, and deformation properties of highly elastic asphalt concrete, the optimal modification method involves blending 3% rubber particles and 1.2% polyester fibers.

When the amount of polyester fiber is not more than 0.6%, the optimal oil–stone ratio is 1:5; when it exceeds 0.6%, the optimal oil–stone ratio is 1:4. The subsequent tests can be carried out with better results according to this mix proportion.

The incorporation of rubber particles reduces the compressive strength of high-elasticity asphalt concrete. Adding an appropriate amount of polyester fibers can increase the compressive strength of high-elasticity asphalt concrete. Appropriate rubber particles and polyester fibers can improve the high-temperature stability, low-temperature crack resistance, and deformation recovery ability of asphalt concrete. Among them, the optimal dosage of polyester fibers is 1.2%, and the optimal dosage of rubber particles is about 2–3%.

Highly elastic asphalt concrete boasts exceptional high- and low-temperature performance, along with superior deformation properties, which fully cater to the demands of bridge structures at frequency and width joints. It extends the service life, reduces the full life cycle cost, realizes effective reuse of resources, reduces environmental pollution, and meets the requirements of green building and circular economy.

In actual projects, where elastic asphalt concrete is used in the construction of pavement under non-stop service conditions, the forces involved are more complex. However, this study focuses solely on the material’s static properties and does not delve into its performance under specific circumstances. To facilitate its future development, it is imperative to undertake further research. This includes investigating the cracking characteristics of elastic asphalt concrete under uneven displacement, simulating its actual force conditions more realistically, and conducting early performance tests of high-elasticity asphalt concrete. Specifically, evaluating the material’s performance when its strength is not fully developed during the construction period can provide valuable insights and protection measures. Moreover, this paper has not addressed the impact of rubber particles or their surface treatment on the performance of asphalt concrete. Research in these areas should also continue. With the continuous progress of technology and the accumulation of application experience, this material will undoubtedly play an increasingly important role in bridge widening and renovation projects, contributing to the construction of a greener, low-carbon, and sustainable world.

## Figures and Tables

**Table 1:** The basic performance indicators of BJ200 asphalt.

Property	Standard	Value
Softening point, °C	JTG E20-2011 [21]	93.8
Ductility (5 °C), cm		14.5
Penetration (25 °C), mm		2.5
Elastic recovery rate, %		75
Viscosity (190 °C), Pa s	JT/T 798-2019 [22]	4.5

**Table 2:** The basic performance indicators of rubber particles.

Property	Combustion Residue, %	Ash, %	Rubber Content, %	Fiber Content, %	Moisture Content, %
Value	37.5	4.5	51	0.5	0.6

**Table 3:** The basic performance indicators of polyester fiber.

Property	Specific Gravity	Elastic Modulus, MPa	Ultimate Elongation, %	Tensile Strength, MPa	Melting Point, °C	Ignition Point, °C
Value	0.91	13.5	9 ± 3	550	259	554

**Table 4:** The design of mix proportion for high-elasticity asphalt concrete.

Name	Oil–Stone Ratio	Bitumen	Aggregates	Rubber Particles	Polyester Fiber
		kg/m^3^	kg/m^3^	kg/m^3^	%
NC	1:5	388	1938	0	0
F0.6	1:5	388	1938	0	0.6
F1.2	1:4	451	1803	0	1.2
F1.8	1:4	451	1803	0	1.8
R2	1:5	388	1899	39	0
R3	1:5	388	1880	58	0
R4	1:5	388	1860	78	0
R2F0.6	1:5	388	1899	39	0.6
R2F1.2	1:4	451	1767	36	1.2
R2F1.8	1:4	451	1767	36	1.8
R3F0.6	1:5	388	1880	58	0.6
R3F1.2	1:4	451	1749	54	1.2
R3F1.8	1:4	451	1749	54	1.8
R4F0.6	1:5	388	1860	78	0.6
R4F1.2	1:4	451	1731	72	1.2
R4F1.8	1:4	451	1731	72	1.8

## Data Availability

Data is contained within the article.
